# Burden of syphilis among people living with HIV: a large cross-sectional study from Türkiye

**DOI:** 10.1007/s10096-025-05199-1

**Published:** 2025-06-24

**Authors:** Selim Merdan, Yağmur Ekenoğlu Merdan, Okan Aydoğan

**Affiliations:** 1 Flow Cytometry Laboratory, Ümraniye Training and Research Hospital, Istanbul, Türkiye; 2 Department of Medical Microbiology, Biruni University School of Medicine, Istanbul, Türkiye; 3 Biruni Research Center, Biruni University, Istanbul, Türkiye; 4 Department of Medical Microbiology, Istanbul Medipol University School of Medicine, Istanbul, Türkiye; 5 Research Institute for Health Sciences and Technologies (SABITA), Istanbul Medipol University, Istanbul, Türkiye

**Keywords:** Syphilis, HIV, Seropositivity, PLWH, STIs

## Abstract

**Background:**

Syphilis and HIV are closely linked infections with shared transmission routes and risk factors. Despite effective screening and treatment options, the prevalence of syphilis remains high among people living with HIV (PLWH), especially in middle-income countries like Türkiye.

**Objectives:**

To investigate the seroprevalence of syphilis among PLWH using centralized laboratory data and to assess its distribution by age and sex.

**Methods:**

A retrospective cross-sectional study was conducted by reviewing data derived from blood specimens submitted to our laboratory for routine CD4⁺ T cell enumeration, ultimately analyzing 2,768 PLWH between January 2022 and December 2024. Syphilis seropositivity was defined by concurrent positivity in both VDRL and TPHA tests. Demographic and clinical variables were analyzed using SPSS v.22.0.

**Results:**

Syphilis seropositivity was detected in 923 patients (33.3%). The prevalence was significantly higher among males (37.0%) compared to females (6.1%) (*p* < 0.001). The highest seroprevalence was observed in patients aged 31–40 years (37.4%). There was no significant difference in mean age between syphilis-positive and syphilis-negative groups (*p* > 0.05).

**Conclusion:**

This study reveals a high burden of syphilis among PLWH in Türkiye, particularly in younger male populations. The findings underscore the necessity for routine syphilis screening, targeted prevention strategies, and integrated STI care within HIV programs.

## Introduction

Syphilis is a sexually and vertically transmitted bacterial infection caused by *Treponema pallidum*. *T. pallidum* is also responsible for non-venereal treponematoses, including yaws, pinta, and bejel (endemic syphilis), which are transmitted through skin-to-skin contact, mainly among children living in warm climates [1]. Although syphilis can be diagnosed at the point of care by rapid lateral flow antibody tests and effectively treated with a single dose of long-acting penicillin, it remains a major cause of adverse pregnancy outcomes in many low- and middle-income countries (LMICs), and its incidence has been rising in high-income countries over the past few decades [1].

According to the most recent World Health Organization (WHO) estimates, approximately 17.7 million adults aged 15 to 49 years were living with syphilis globally in 2012, with an estimated 6.3 million new cases in 2016 [2, 3]. The prevalence and incidence varied substantially by region, with the highest rates in Africa and over 60% of new diagnoses occurring in LMICs. While high-income countries maintain low syphilis prevalence among heterosexual men and women, a notable resurgence has been documented among men who have sex with men (MSM), closely linked to Human Immunodeficiency Virus (HIV) infection and high-risk sexual behaviors [4].

HIV infection remains a major global health concern and is frequently associated with an increased risk of sexually transmitted infections (STIs), particularly syphilis. Moreover, recent evidence highlights the complex syndemic interactions between HIV, syphilis, other STIs, and emerging infections, which may further complicate clinical management and prevention strategies among PLWH [5–7]. Globally in 2023, 74% of reporting countries (91 out of 123) had established national plans for the elimination of vertical transmission of both HIV and syphilis, with most adopting integrated strategies. An additional 17% of countries had plans targeting either HIV or syphilis elimination individually. WHO promotes a comprehensive approach based on four pillars: primary prevention of infection and transmission, integration of sexual and reproductive health services, core maternal services, and services for infants, children, and partners. To date, 17 countries or territories have been validated for eliminating vertical transmission of both infections [8]. These achievements underscore the global recognition of the intertwined epidemiology of HIV and syphilis and highlight the necessity for ongoing surveillance and targeted interventions.

Syphilis has exerted a substantial impact on several high-risk populations throughout history. Since 2000, the rates of primary and secondary syphilis in the United States have risen sharply, predominantly driven by a more than threefold increase among men. In 2018, men accounted for 86% of all syphilis cases, with more than half reporting sex with men. Among these, 42% were co-infected with HIV, reinforcing the strong epidemiological link between syphilis and HIV acquisition [4, 9, 10]. Similar trends have been reported in Europe and China, where the incidence of syphilis among MSM populations has also increased [11, 12]. These patterns emphasize the bidirectional relationship between HIV and syphilis, underscoring the importance of integrating STI surveillance and preventive efforts within HIV care programs [4, 13].

Reliable diagnosis of HIV infection is a cornerstone of integrated prevention and care strategies. Advances in point-of-care nucleic acid testing and molecular diagnostics, such as quantitative PCR, have greatly enhanced early HIV detection and monitoring, contributing to improved outcomes and epidemic control [14].

CD4 + T cells play a central role in the immunopathogenesis of HIV infection and in the progression of disease. During HIV replication, CD4 + T cells undergo massive depletion due to both direct viral cytopathic effects and immune-mediated mechanisms, ultimately leading to immune deficiency [15] In addition, molecular pathways involved in restricting HIV infection in CD4 + T cells, such as the action of MCPIP1, have also been described [16].

Despite these concerning trends, most existing studies on syphilis among PLWH have been limited to specific populations, small sample sizes, or single-center designs. Particularly in regions such as Türkiye, where the HIV epidemic is evolving, large-scale, multi-center data regarding the syphilis burden remain scarce. Understanding the epidemiology of syphilis in this context is critical for informing public health strategies, optimizing screening protocols, and improving clinical management. In this study, we aimed to evaluate the seroprevalence of syphilis among a large study group of PLWH by utilizing centralized laboratory data spanning multiple healthcare institutions. We further sought to characterize the distribution of seropositivity across sex and age groups, thereby providing insights into the demographic patterns of syphilis infection in this vulnerable population.

## Materials and methods

**Fig. 1:**
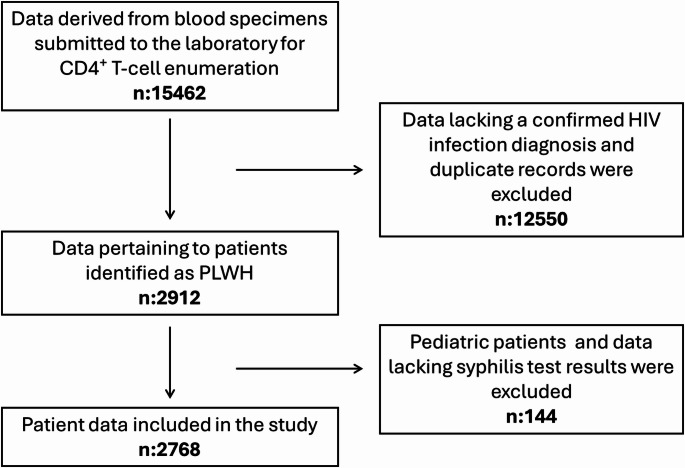
Flow diagram of the sampling process

Ethical approval for this study received from the Ümraniye Training and Research Hospital Ethics Committee on 27 February 2025 (Approval No. 06). We conducted a cross-sectional retrospective study utilizing patient data derived from blood specimens sent to our central flow cytometry laboratory, which serves eight hospitals in the city and performs a variety of flow cytometry tests, for CD4 + T-cell enumeration between January 2022 and December 2024. During this period, a total of 15,462 sample records were screened; duplicate entries were removed, and records from patients without a confirmed HIV diagnosis were excluded, leaving 2,912 unique records for PLWH; after the removal of pediatric cases (*n* = 3) and records without syphilis results (*n* = 141), 2,768 unique patient records were left for analysis. A total of 2,768 PLWH were evaluated. Patients with both VDRL and TPHA negative (1,755 patients), unknown VDRL but negative TPHA (4 patients), and VDRL positive with TPHA negative (86 patients) were considered negative for syphilis. Patients with both VDRL and TPHA positive (923 patients) were considered positive for syphilis. No individuals were identified as VDRL-negative with a TPHA-positive result. A flow diagram detailing the sampling process is presented in Fig. 1.

For each patient, we recorded age, sex, and syphilis serostatus. To examine age-related trends, participants were divided into eight groups: 18–20, 21–30, 31–40, 41–50, 51–60, 61–70, 71–80 and 81–90 years. All personal identifiers were irreversibly anonymized to ensure confidentiality.

Syphilis serostatus was established by applying both the traditional (non-treponemal–first) and reverse (treponemal–first) serological algorithms. In the traditional algorithm, initial screening is done with the non-treponemal Venereal Disease Research Laboratory (VDRL) test; specimens yielding a reactive VDRL are then confirmed by the treponemal Treponema pallidum hemagglutination assay (TPHA). Conversely, in the reverse algorithm, initial screening is done with TPHA, and any TPHA-reactive samples were subsequently tested by VDRL; specimens reactive on TPHA but non-reactive on VDRL were interpreted as indicative of past or latent infection. Serologically confirmed syphilis was defined as having a reactive RPR as well as a reactive treponemal test upon study enrollment. Seropositivity was assigned only when both VDRL and TPHA yielded concurrent positive results, thereby confirming treponemal exposure. Conversely, a seronegative status was designated in all other cases, namely: [1] both VDRL and TPHA negative; [2] VDRL negative with unknown TPHA; [3] TPHA negative with unknown VDRL; and [4] VDRL positive but TPHA negative (interpreted as a biological false positive).

Statistical analyses were performed using Statistical Package for Social Sciences v.22.0 (IBM Corp.; Armonk, NY, USA). Patient demographics and syphilis serostatus were summarized as absolute numbers and percentages. Syphilis seroprevalence was stratified by predefined age categories and by biological sex. Differences in seropositivity across age groups and between males and females were assessed using Pearson’s chi-square test; when expected cell counts were < 5, Fisher’s exact test was applied. A two-tailed p-value < 0.05 was considered statistically significant.

## Results

**Fig. 2:**
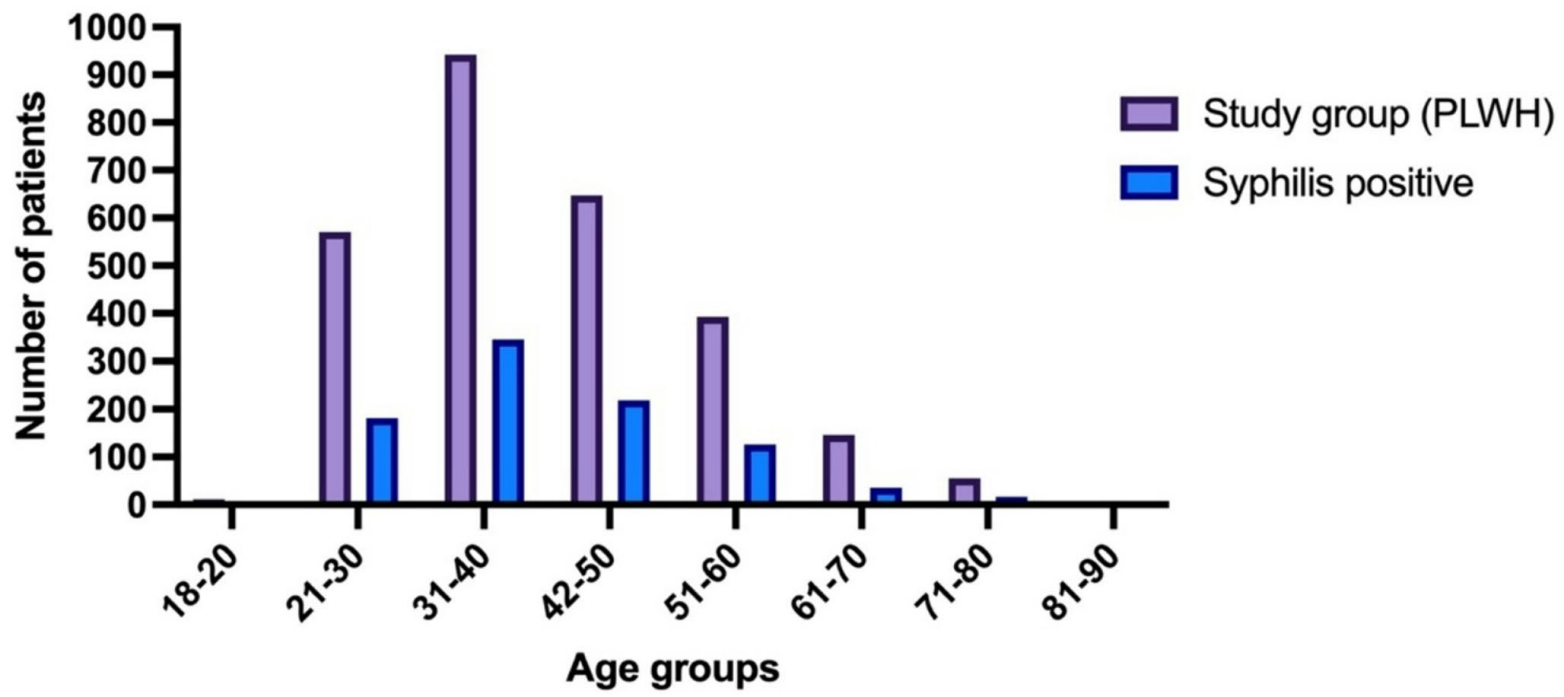
Age-stratified distribution of the study population and syphilis-positive cases among people living with HIV (PLWH)

Of the 2,768 PLWH included in the analysis, 326 (11.8%) were female and 2442 (88.2%) were male. The overall mean age was 40.9 ± 12.2 years (range, 18–86 years). Female patients had a mean age of 44.5 ± 12.1 years (range, 19–80 years), whereas male patients had a mean age of 40.4 ± 12.1 years (range, 18–86 years). Among the study group, 1,009 individuals (36.4%) had a reactive VDRL result, and 923 of these individuals (91.5%) were subsequently confirmed seropositive by TPHA testing. The seroprevalence of syphilis varied significantly among different age groups (*p* = 0.015, Pearson’s chi-square = 17.418, df = 7). The highest rates were observed in patients aged 31–40 years (37.4%), followed by those aged 41–50 years (23.7%) and 21–30 years (19.6%). The mean age of patients with serologically confirmed syphilis was 40.3 ± 11.3 years (range, 21–86 years). The age-group-specific distribution of syphilis seropositivity is presented in Fig. 2. Of these 923 seropositive patients, 20 were female (2.2%) and 903 were male (97.8%). The seroprevalence was significantly higher among males (37.0%) compared to females (6.1%) (*p* < 0.001, Pearson’s chi-square = 123.096). The gender and age-group-specific distribution of syphilis seropositivity is presented in Table 1. The overall syphilis seroprevalence in the study group was 33.3% (95% CI: 31.5–35.1%). Among male patients, the prevalence was 37.0% (95% CI: 35.0–39.1%), while among female patients, it was 6.1% (95% CI: 3.7–9.3%).

The mean age of syphilis-positive patients (40.3 ± 11.3 years) did not differ significantly from that of syphilis-negative patients (41.2 ± 12.4 years, *p* > 0.05).

**Table 1:** The gender and age-group-specific distribution of syphilis seropositivity

Age Group	Female (*n*)	Syphilis-Positive Female (*n*, % of females in group)	Male (*n*)	Syphilis-Positive Male (*n*, % of males in group)
18–20	3	0 (0.0%)	8	0 (0.0%)
21–30	44	3 (6.8%)	526	178 (33.8%)
31–40	94	6 (6.4%)	848	339 (40.0%)
41–50	85	4 (4.7%)	562	215 (38.3%)
51–60	59	6 (10.2%)	334	120 (35.9%)
61–70	32	1 (3.1%)	114	34 (29.8%)
71–80	9	0 (0.0%)	46	16 (34.8%)
81–90	0	0 (0.0%)	4	1 (25.0%)
**Total**	326	20 (6.1%)	2442	903 (37.0%)

*p < 0.001 by chi-square test comparing overall male vs. female seroprevalence

Syphilis seroprevalence in males: 37.0% (95% CI: 35.0–39.1%); in females: 6.1% (95% CI: 3.7–9.3%)

## Discussion

Türkiye has experienced a significant rise in HIV cases in recent years. According to national data, 45,835 people were documented as PLWH in Türkiye from 1985 to 2024, with a notable increase in incidence observed over the last decade [16]. The WHO reports a 45% increase in new HIV infections since 2015 in the Eastern Mediterranean region, counter to the global declining trend. This surge provides context for evaluating co-infections with other STIs like syphilis [17]. Syphilis seropositivity is a growing concern in Turkish HIV-positive populations. Local studies indicate syphilis co-infection rates varying from about 7–20% among PLWH, depending on the region and risk group [10, 17–22].

In a cohort from southern Türkiye, 7.6% of HIV-positive patients had syphilis co-infection, with nearly all co-infected cases being male (median age 33) [22]. In Türkiye, a 2018 study found an overall syphilis seroprevalence of 19.3% among HIV-positive men, with MSM particularly affected (28.7% syphilis prevalence in MSM vs. much lower in heterosexual men) [21]. This high rate among MSM underscores the overlapping sexual risk networks of HIV and syphilis transmission. Although detailed national syphilis surveillance data for Türkiye are limited, the increase in syphilis among PLWH reflects a broader re-emergence of syphilis. Our study, conducted in İstanbul, the most populous metropolis in both Türkiye and Europe, offers a valuable representation of HIV related syphilis epidemiology in a setting whose demographic scale amplifies the public health relevance of the findings.

The co-occurrence of HIV and syphilis is epidemiologically significant due to a syndemic relationship. Syphilis, a genital ulcerative disease, can facilitate HIV transmission by breaching mucosal barriers. In turn, having HIV (especially if untreated) may predispose to more frequent or severe syphilis infections [1, 12, 13, 21, 23]. Individuals with syphilis are at higher risk of acquiring HIV, and HIV-negative MSM diagnosed with syphilis are more likely to seroconvert to HIV in the future. Conversely, syphilitic sores increase the likelihood of HIV transmission to partners. This bidirectional enhancement means that rises in one infection can fuel the other, creating a dual epidemic in high-risk networks.

Syphilis seropositivity is significantly higher among PLWH compared to the general population and has shown an increasing trend [1, 12, 13, 21, 23]. In our study group, syphilis seropositivity was identified in 33.4% of 2,768 PLWH, indicating that approximately one in every three PLWH had either a past or current syphilis infection. This proportion has reflected the shared risk factors and transmission routes of the two infections.

Epidemiological data indicate that syphilis cases have been rising in communities where the HIV epidemic is prevalent. In particular, key populations such as MSM have experienced a marked increase in syphilis incidence over the past decade [21]. In Europe, syphilis cases have increased significantly in the last ten years, with the main proportion of new diagnoses occurring in PLWH [8, 12, 24].

From a demographic perspective, as observed in our study, syphilis coinfection among PLWH tends to be concentrated within specific subgroups. The majority of coinfected patients were male, while female patients remained a clear minority in this cohort. Age distribution shows that coinfected individuals typically fall within the young-to-middle adulthood range. In our study, the highest syphilis seropositivity was detected in the 31–40 age group. This finding shows that the greatest risk is among sexually active young adult men. Indeed, when assessed by risk group, syphilis seroprevalence is markedly higher among HIV-positive MSM or bisexual individuals compared to their heterosexual counterparts [9]. This discrepancy suggests that unprotected sexual practices and overlapping sexual networks in MSM communities substantially facilitate the transmission of syphilis.

In the literature, cases of recurrence or treatment failure of syphilis have been reported among PLWH, even after appropriate therapy [9, 25]. The current guidelines recommend more frequent serologic follow-up, such as RPR testing every three months, after treatment in PLWH with syphilis [26]. An unintended consequence of advancements in HIV treatment is the observed complacency in protective sexual behaviors. The “Undetectable = Untransmittable (U = U)” concept represents a major breakthrough in HIV/AIDS management [27]. However, the widespread adoption of this message has, in some cases, led to a deprioritization of condom use [28]. While confidence in preventing HIV transmission is well-founded, it does not offer protection against other STIs, such as syphilis. Indeed, declining condom use has been cited as one of the contributing factors to the global resurgence of syphilis among HIV-positive populations [28]. Therefore, clinical follow-up for PLWH should emphasize the need for protection not only against HIV but also against other STIs. In particular, sustained sexual health education is essential for younger PLWH who remain healthy for extended periods under treatment, as even a “treatable” infection like syphilis can lead to serious complications if neglected.

For instance, a large-scale meta-analysis conducted in China estimated the overall syphilis seropositivity rate among PLWH to be approximately 18% [8]. Similarly, the coexistence of syphilis and HIV is highly prevalent in major urban centers across Europe and North America, particularly among MSM, where both infections are disproportionately concentrated. According to ECDC, in certain European countries, one-third or more of MSM diagnosed with syphilis are also living with HIV [2, 3, 20, 23]. These striking co-infection rates suggest that syphilis has become an almost ‘accompanying’ infection within populations heavily affected by the HIV epidemic.

The high syphilis seropositivity rate identified in our study may be attributed to the demographic and behavioral characteristics of our study population. In particular, the elevated proportion of MSM individuals, the urban-centered nature of the cohort, and the presence of common risk factors for sexually transmitted infections among patients may account for this finding. These findings suggest that Türkiye is not exempt from global trends in HIV-syphilis coinfection; on the contrary, it faces similar public health challenges.

The high burden of syphilis seropositivity among PLWH underscores the need for a comprehensive, multifaceted public health response. This necessitates the implementation of routine and targeted screening programs, timely and effective therapeutic interventions, continuous sexual health education, and tailored policies addressing key populations at elevated risk. It is imperative to conceptualize the HIV and syphilis epidemics as interconnected and synergistic rather than independent entities. Such an integrated framework may enhance the effectiveness of prevention efforts, facilitate early detection of co-infections, and ultimately improve health outcomes in this vulnerable population.

### Limitations

This study has several limitations. First, the retrospective nature and reliance on centralized laboratory data precluded access to behavioral risk factors, treatment history, or clinical outcomes. Second, as the data lacked temporal stratification, trends over time in syphilis incidence could not be evaluated. Because the dataset comprised only specimens submitted for routine CD4⁺ T cell monitoring, PLWH who did not have a CD4 count requested during the study period, those not yet linked to care, or lost to follow-up, were not captured in the analysis. Despite these limitations, the large sample size and standardized testing protocols enhance the robustness and generalizability of the findings. We acknowledge that defining syphilis seropositivity based only on concurrent VDRL and TPHA positivity may potentially underestimate the true prevalence, as latent or previously treated cases with discordant results could have been missed. Moreover, multivariable analyses could not be performed due to the unavailability of behavioral and clinical risk factor data in the retrospective dataset.

## Conclusion

Our findings reveal a strikingly high seroprevalence of syphilis among PLWH in Türkiye, underscoring the intertwined nature of these two epidemics. The observed co-infection rate not only exceeds global averages but also highlights critical gaps in prevention strategies. In light of these results, integrated screening, targeted education, and sustained public health interventions are urgently needed, especially among high-risk subpopulations. Future multicenter and longitudinal studies are essential to monitor trends, evaluate treatment outcomes, and optimize response strategies at both national and regional levels.

## Data Availability

No datasets were generated or analysed during the current study.

## References

[1] Peeling RW, Mabey D, Chen XS, Garcia PJ (2023) Syphilis. The Lancet [Internet]. Jul 22 [cited 2025 Apr 27];402(10398):336–46. Available from: https://pubmed.ncbi.nlm.nih.gov/37481272/10.1016/S0140-6736(22)02348-0 37481272

[2] WHO. Global Health Sector Strategies on HIV, Viral Hepatitis and the sexually transmitted infections. World Health Organization [Internet] (2021) [cited 2025 Apr 27];ector stra(June 2021):2022–30. Available from: https://www.who.int/publications/i/item/9789240027077

[3] Rowley J, Hoorn S, Vander, Korenromp E, Low N, Unemo M, Abu-Raddad LJ et al Chlamydia, gonorrhoea, trichomoniasis and syphilis: Global prevalence and incidence estimates, 2016. Bull World Health Organ [Internet]. 2019 Aug 1 [cited 2025 Apr 27];97(8). Available from: https://pubmed.ncbi.nlm.nih.gov/31384073/10.2471/BLT.18.228486 PMC6653813 31384073

[4] Ghanem KG, Ram S, Rice PA The Modern Epidemic of Syphilis. New England Journal of Medicine [Internet]. 2020 Feb 27 [cited 2025 Apr 27];382(9):845–54. Available from: https://pubmed.ncbi.nlm.nih.gov/32101666/10.1056/NEJMra1901593 32101666

[5] Elendu C, Amaechi DC, Elendu ID, Elendu TC, Amaechi EC, Usoro EU et al Global perspectives on the burden of sexually transmitted diseases: A narrative review. Medicine (United States) [Internet]. 2024 May 17 [cited 2025 Jun 8];103(20):E38199. Available from: https://journals.lww.com/md-journal/fulltext/2024/05170/global_perspectives_on_the_burden_of_sexually.37.aspx 10.1097/MD.0000000000038199 PMC11098264 38758874

[6] Mody A, Sohn AH, Iwuji C, Tan RKJ, Venter F, Geng EH HIV epidemiology, prevention, treatment, and implementation strategies for public health. Lancet [Internet]. 2024 Feb 3 [cited 2025 Jun 8];403(10425):471–92. Available from: https://pubmed.ncbi.nlm.nih.gov/38043552/10.1016/S0140-6736(23)01381-8 38043552

[7] Bekker LG, Beyrer C, Mgodi N, Lewin SR, Delany-Moretlwe S, Taiwo B et al HIV infection. Nat Rev Dis Primers [Internet]. 2023 Dec 1 [cited 2025 Jun 8];9(1). Available from: https://pubmed.ncbi.nlm.nih.gov/37591865/10.1038/s41572-023-00452-3 37591865

[8] 2024 global AIDS report — The Urgency of Now AIDS at a Crossroads| UNAIDS [Internet]. [cited 2025 Apr 27]. Available from: https://www.unaids.org/en/resources/documents/2024/global-aids-update-2024

[9] Doron A, Rahav G, Wieder-Feinsod A, Litchevski V, Olmer L, Amit S, AIDS [Internet] (2022) Syphilis reinfection among people living with HIV. Int J STD. Mar 1 [cited 2025 Apr 27];33(4):416–7. Available from: https://pubmed.ncbi.nlm.nih.gov/35077250/10.1177/09564624211069016 35077250

[10] Sarıgül F, Sayan M, İnan D, Deveci A, Ceran N, Çelen MK et al Current status of HIV/AIDS-syphilis co-infections: A retrospective multicentre study. Cent Eur J Public Health [Internet]. 2019 Sep 1 [cited 2025 Apr 27];27(3):223–8. Available from: https://pubmed.ncbi.nlm.nih.gov/31580558/10.21101/cejph.a5467 31580558

[11] Zeng Q, Yang Y, Zhang L, Yan J, Wang J, Nie J et al The impact of the National Syphilis Prevention Program on the prevalence of syphilis among people living with HIV in China: a systematic review and meta-analysis. J Int AIDS Soc [Internet]. 2025 Jan 1 [cited 2025 Apr 27];28(1). Available from: https://pubmed.ncbi.nlm.nih.gov/39763073/10.1002/jia2.26408 PMC11705538 39763073

[12] Janier M, Unemo M, Dupin N, Tiplica GS, Potočnik M, Patel R (2020) European guideline on the management of syphilis. Journal of the European Academy of Dermatology and Venereology [Internet]. 2021 Mar 1 [cited 2025 Apr 27];35(3):574–88. Available from: https://pubmed.ncbi.nlm.nih.gov/33094521/10.1111/jdv.16946 33094521

[13] Gilmour LS, Walls T (2023) Congenital Syphilis: a Review of Global Epidemiology. Clin Microbiol Rev [Internet]. Jun 1 [cited 2025 Apr 27];36(2). Available from: https://pubmed.ncbi.nlm.nih.gov/36920205/10.1128/cmr.00126-22 PMC10283482 36920205

[14] Pinar SS, Manak M, Saravanan S, Imami N, Kibirige C Point-of‐care nucleic acid testing– a step forward in controlling the HIV epidemic: A review. HIV Med [Internet]. 2025 Apr 1 [cited 2025 Jun 8];26(4):536. Available from: https://pmc.ncbi.nlm.nih.gov/articles/PMC11970346/10.1111/hiv.13757 PMC11970346 39865395

[15] Doitsh G, Galloway NLK, Geng X, Yang Z, Monroe KM, Zepeda O et al (2014) Cell death by pyroptosis drives CD4 T-cell depletion in HIV-1 infection. Nature 505(7484):509–514 24356306 10.1038/nature12940 PMC4047036

[16] Dokümanlar [Internet] [cited 2025 May 5]. Available from: https://hsgm.saglik.gov.tr/tr/dokumanlar-bulasicihastaliklar.html

[17] Baltalı S, Soytürk AN, Sarı DN (2024) Evaluation of Intensive Care Patients: A Single-center Experience. Bagcilar Medical Bulletin [Internet]. 2024 Nov 11 [cited 2025 May 4]; Available from: https://www.behmedicalbulletin.org/articles/evaluation-of-intensive-care-patients-a-single-center-experience/doi/BMB.galenos.2024-07-059

[18] Mustafayev K, Mete B, Kutlubay Z, Tanakol A, Şahin Özdemir M, Garashova D, AIDS [Internet] Dermatological lesions among people living with HIV in Turkey. Int J STD. 2022 Jan 1 [cited 2025 May 5];33(1):55–62. Available from: https://pubmed.ncbi.nlm.nih.gov/34565234/10.1177/09564624211043711 34565234

[19] Aydın ÖA, Kumbasar Karaosmanoğlu H, Sayan M, İnce ER, Nazlıcan Ö Seroprevalence and risk factors of syphilis among HIV/AIDS patients in Istanbul, Turkey. Cent Eur J Public Health [Internet]. 2015 [cited 2025 May 5];23(1):65–8. Available from: https://pubmed.ncbi.nlm.nih.gov/26036101/10.21101/cejph.a4001 26036101

[20] Gökengin D, Noori T, Alemany A, Bienkowski C, Liegon G, İnkaya AÇ et al Prevention strategies for sexually transmitted infections, HIV, and viral hepatitis in Europe. The Lancet Regional Health - Europe [Internet]. 2023 Nov 1 [cited 2025 May 5];34. Available from: https://pubmed.ncbi.nlm.nih.gov/37927439/10.1016/j.lanepe.2023.100738 PMC10625023 37927439

[21] Köksal MO, Beka H, Evlice O, Çiftçi S, Keskin F, Başaran S et al (2020) Syphilis seroprevalence among HIV-infected males in Istanbul, Turkey. Rev Argent Microbiol [Internet]. Oct 1 [cited 2025 May 5];52(4):266–71. Available from: https://pubmed.ncbi.nlm.nih.gov/32178940/10.1016/j.ram.2020.01.002 32178940

[22] Kömür S, Ertürk D, Sevdimbaş S, Kuşcu F, İnal AS, Kurtaran B et al Evaluation of HIV and Syphilis Co-infected Cases, Data from a University Hospital. Curr HIV Res [Internet]. 2024 May 17 [cited 2025 May 5];22(3):153–7. Available from: https://pubmed.ncbi.nlm.nih.gov/38757313/10.2174/011570162X313718240514042111 38757313

[23] Adawiyah R, Al, Saweri OPM, Boettiger DC, Applegate TL, Probandari A, Guy R et al The costs of scaling up HIV and syphilis testing in low-and middle-income countries: A systematic review. Health Policy Plan [Internet]. 2021 Jul 1 [cited 2025 Apr 27];36(6):939–54. Available from: https://pubmed.ncbi.nlm.nih.gov/33693731/10.1093/heapol/czab030 PMC8227996 33693731

[24] Wu MY, Gong HZ, Hu KR, Zheng HY, Wan X, Li J Effect of syphilis infection on HIV acquisition: a systematic review and meta-analysis. Sex Transm Infect [Internet]. 2021 Nov 1 [cited 2025 May 5];97(7):525–33. Available from: https://sti.bmj.com/content/97/7/525 10.1136/sextrans-2020-054706 PMC8543214 33219164

[25] Lin KY, Yang CJ, Sun HY, Chuang YC, Chang LH, Liu WC et al Comparisons of Serologic Responses of Early Syphilis to Treatment with a Single-Dose Benzathine Penicillin G Between HIV-Positive and HIV-Negative Patients. Infect Dis Ther [Internet]. 2021 Sep 1 [cited 2025 May 5];10(3):1287. Available from: https://pmc.ncbi.nlm.nih.gov/articles/PMC8322187/10.1007/s40121-021-00450-6 PMC8322187 33948910

[26] STI Screening Recommendations [Internet] [cited 2025 May 5]. Available from: https://www.cdc.gov/std/treatment-guidelines/screening-recommendations.htm

[27] Bekker LG, Smith P, Ntusi NAB (2023) HIV is sexually untransmittable when viral load is undetectable. The Lancet [Internet]. Aug 5 [cited 2025 May 5];402(10400):428–9. Available from: https://www.thelancet.com/action/showFullText?pii=S0140673623015192 10.1016/S0140-6736(23)01519-2 37490934

[28] Hixson LK, Drach L, Maher JE, Knapp AT, Ferrer JS, Menza TW Factors Associated with Increased Syphilis Screening among People Living with Human Immunodeficiency Virus. Sex Transm Dis [Internet]. 2019 Aug 1 [cited 2025 May 5];46(8):521–6. Available from: https://pubmed.ncbi.nlm.nih.gov/31295220/10.1097/OLQ.0000000000001015 31295220

